# School-based interventions targeting double burden of malnutrition and educational outcomes of adolescents in low- and middle-income countries: protocol for a systematic review

**DOI:** 10.1186/s13643-021-01756-9

**Published:** 2021-07-10

**Authors:** Sachin Shinde, Dongqing Wang, Wafaie W Fawzi

**Affiliations:** 1 Department of Global Health and Population, Harvard T.H. Chan School of Public Health, 3rd Floor, 90 Smith Street, Boston, MA 02120 USA; 2 Center for Inquiry into Mental Health, Pune, India; 3 Department of Epidemiology, Harvard T.H. Chan School of Public Health, Boston, MA USA; 4 Department of Nutrition, Harvard T.H. Chan School of Public Health, Boston, MA USA

**Keywords:** Double burden of malnutrition, Adolescents, Low − and middle − income countries, Systematic review, Randomized controlled trials, Controlled before-after studies

## Abstract

**Background:**

Adolescence is a period of rapid physical growth and transition between childhood to adulthood. However, in many developing countries, nutritional and epidemiological transitions are contributing to surging overnutrition, which, together with prevalent undernutrition, is resulting in the double burden of malnutrition (DBM) among adolescents. Schools as social systems have tremendous but mostly underutilized capacity to facilitate change and address a range of nutritional and associated educational concerns of adolescents and young people. The main objective of this systematic review will be to describe school-based interventions that address the multiple forms of malnutrition, and synthesize their effects on nutrition and educational outcomes among adolescents (10 − 19 − years − old) from low- and middle-income countries (LMICs).

**Methods:**

Comprehensive literature searches will be conducted in multiple electronic databases, including Medline (through PubMed), Embase, CENTRAL (through Cochrane Library), CINAHL, and Google Scholar. We will include randomized controlled trials (RCTs), non-RCTs including controlled before-after studies, examining the effects of nutrition interventions on nutrition and educational outcomes among adolescents in LMICs. Two reviewers will independently screen all citations and full-text articles and abstract data. The quality of the included studies will be assessed with the Cochrane Collaboration’s revised tool for assessing the risk of bias for RCTs and the Risk Of Bias In Non-randomized Studies of Interventions tool for controlled before-after studies and non-randomized controlled trials.

**Discussion:**

To maximize the power of schools as a platform to reinforce the mutually beneficial relationship between adolescent nutrition and education, it is imperative to develop and implement integrated interventions connecting schools, adolescents, parents, communities, and the health care system. The results of this systematic review may provide a comprehensive state of current knowledge on the effectiveness of school-based interventions to enable future research that maximizes the impact and efficiency of integrated approaches to tackle multiple forms of malnutrition among school-going and out-of-school adolescents.

**Systematic review registration:**

PROSPERO ID: CRD42020211109

**Supplementary Information:**

The online version contains supplementary material available at 10.1186/s13643-021-01756-9.

## Background

Approximately 1.2 billion adolescents aged 10–19 years today make up 16% of the world’s population, of which 90% live in low- and middle-income countries (LIMCs) [1]. Over 340 million children and adolescents were overweight or obese across the world in 2016 [2]. Around 240 million children and adolescents were overweight (18% girls and 19% boys), while more than 124 million children and adolescents (6% of girls and 8% of boys) were obese. The prevalence of childhood and adolescent underweight has decreased globally, from 37.0% in 2000 to 31.6% in 2016 among boys, and from 29.6% in 2000 to 25.9% in 2016 among girls [2, 3]. The Global School-based Student Health and Health Behaviour Survey between 2003 and 2013, in 129, 276 school-aged adolescents (aged 12 − 15 years) from 57 LMICs estimated the prevalence of stunting as 10.2%, the prevalence of thinness as 5.5%, the prevalence of overweight or obesity as 21.4% and the prevalence of concurrent stunting and overweight or obesity as 2.0% [4].

Globally, there is an unprecedented increase in the coexistence of undernutrition along with overweight and obesity, or diet-related non-communicable diseases, within individuals, households, and populations, and across the life course, giving rise to the term “double burden of malnutrition” (DBM) [5]. The consequences of concurrent undernutrition and overnutrition in adolescents are associated with delayed onset of puberty, poor cognitive development, poor academic performance, and early onset of adult chronic diseases (e.g., type 2 diabetes, and hypertension) [6]. Consecutive undernutrition and overnutrition in adults are associated with less muscle strength, decreased bone density, poor work capacity, and poor reproductive outcomes, particularly among women due to an increased risk of pregnancy [7]. In this perspective, changes in diet and health behaviors are likely to have major effects on the individual’s current and future health [7, 8].

Education is one of the most powerful determinants of adolescent health and a driver of economic progress to a successful transition to adulthood [8]. Given the bidirectional relationship between health and nutrition on the one hand, and educational outcomes on the other [8], schools offer a promising platform for addressing nutritional issues of adolescents. Furthermore, schools and peers have a central role in adolescents’ social lives, thus influencing multiple health behaviors, diet habits, and educational outcomes [8]. This is particularly the case given the global expansion of school attendance [9]. Because schools are at the heart of all communities, there is an opportunity to use the school as a sustainable, scalable option to reinforce health messages and address all forms of malnutrition for both school-going and out-of-school adolescents.

Several systematic reviews suggest promising but modest evidence of benefit from addressing malnutrition through discrete school − based nutrition interventions (e.g., nutrition education, physical activity, and micronutrients through fortification and targeted supplementation, school feeding, school gardens, and access to a safe environment and hygiene) [10–20]. For example, nutrition education, promoting healthy diets, food supplementations and/or fortification, and nutrient supplementation interventions are effective in reducing micronutrient deficiencies and can improve nutrition status [10, 13, 18]. Similarly, lifestyle interventions including dietary interventions, physical activity, and food environment interventions may reduce the risk of overweight and obesity [12–16]. However, these single–domain interventions target either undernutrition or overnutrition and function in *silos*. Hence, there is increasing interest in schools addressing health and nutrition behaviors through integrated interventions, generally called “double-duty actions” [21], to target multiple forms of malnutrition and non-communicable diseases. An essential element of this notion is that addressing one form of malnutrition should not be detrimental to subsequently tackle another form of malnutrition. Evidence on integrated interventions is promising in improving the nutritional status of school-going children and adolescents. However, it comes largely from high-income countries and therefore has limited generalizability [22–29].

Therefore, the purpose of this work is to comprehensively review the literature to describe school-based interventions that address the multiple forms of malnutrition of adolescents (aged 10 to 19 years) in LMICs and describe their effects on nutrition and educational outcomes.

### Conceptual framework

The newly developed agenda for Sustainable Development 2030 has recognized a need for greater accountability especially for the Global Strategy for Women’s, Children’s and Adolescents’ Health [30]. It has called for increased participatory frameworks across a range of areas relevant to young people including non-communicable disease risks and nutrition. We developed a conceptual framework based on existing frameworks of school-based interventions [30, 31] and the recommendations including the Lancet’s 2008 Maternal and Child Undernutrition series [32] and 2013 Maternal and Child Nutrition series [33]. Our framework enlists the underlying causes of malnutrition as household food insecurity, unhealthy diets, inadequate feeding and care practices, sedentary lifestyles, and environmental factors including lack of access to clean drinking water, general hygiene and sanitation practices, household socio-economic status, and lack of access to health services.

**Fig. 1:**
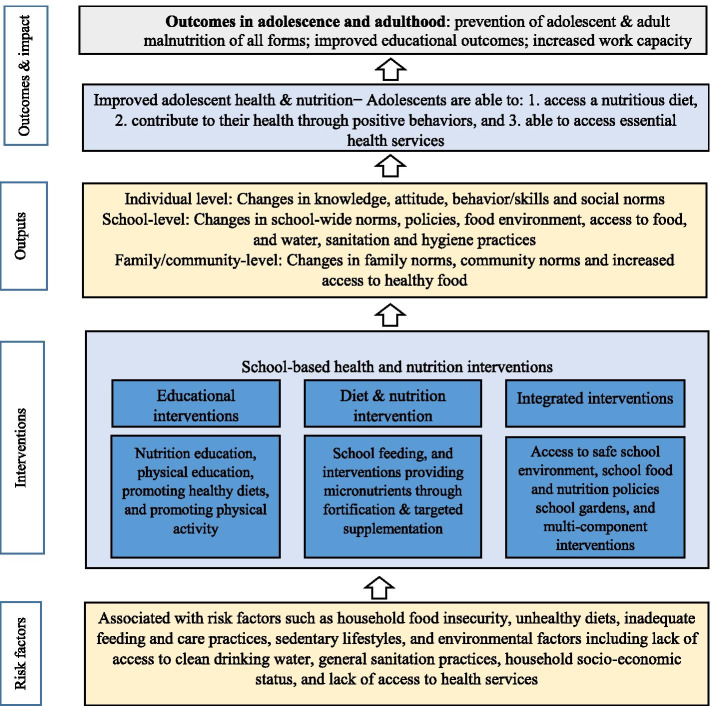
Framework for evidence synthesis of school-based interventions addressing multiple forms of malnutrition among adolescents

Conversely, for adolescents to enjoy health and well-being and improved nutrition, they should have the following minimum capacity or agency: knowledge of healthy diet and nutrition, they are able to access a nutritious diet, they are able to contribute to their health through positive behaviors, and they are able to access essential health services. In terms of implementing interventions to address all forms of malnutrition in adolescents, the school setting often targets one or more elements including school curriculum, food and nutrition environment, school nutrition and health services, school environment. Based on the underlying causes of malnutrition and their corresponding solutions, we identified three broad categories of evidence-based nutrition interventions that could affect adolescent nutrition (Fig. 1).

Outputs associated with these interventions include changes in knowledge, attitudes and behaviors about healthy diets at the individual level; changes in norms, policies and food environment at the school level; and changes in norms and access to healthier foods at the family and community level. These changes would then influence behaviors related to diet, hygiene, cooking and food hygiene skills, as well as concentration and participation in the school environment. These behavior changes would ultimately result in desirable health and educational outcomes, such a reduction of all forms of malnutrition and improved academic performance, among others.

## Methods/design

### Aims of the Review

We aim to address the following points through the review of school-based interventions targeting multiple forms of malnutrition of adolescents in LMICs.

1. Map the interventions that have been carried out
2. Ascertain the effectiveness of these interventions in addressing specific outcomes of interest

The inclusion/exclusion criteria are outlined below and summarized in PICOS format (population, interventions, comparisons, outcomes, and study design) in Table 1.

**Table 1:** Eligibility criteria for the systematic review in PICO format

Item	Inclusion criteria	Exclusion criteria
Population	Adolescents (10–19 years old) in primary and/or secondary school	Studies involving school-age children (10–19 years old) but interventions applied exclusively outside of a school setting
Interventions, approaches or exposures of interests	Studies involving one or more of the following interventions: School-based nutrition and physical education, promoting healthy diets and/or physical activity, school food and nutrition policies, Access to safe school environment, school garden, and water, sanitation and hygiene interventions	Interventions targeted towards individuals with specific medical conditions such as treatments intended for underweight, overweight or obese adolescents; and educational interventions focusing only on educational outcomes (e.g., school or classroom performance, classroom environment,, and teacher performance) Studies of school feeding and nutrient supplementation interventions
Comparison	Studies that compared the intervention with any relevant control group including comparisons with no intervention, regular nutrition education classes and/or physical education classes, or any other intervention in the school setting	Not applicable
Outcomes	Primary outcomes: BMI z score and school attendance Secondary outcomes: a change in anthropometry (e.g., height and weight status, BMI, height-for-age z scores, weight-for-age z scores, weight-for-height z scores, skin-fold thickness measures, stunting, underweight, wasting, body mass index, overweight, obesity, waist-to-height ratio, and central obesity), knowledge of diet and nutrition, dietary intake (i.e. amount and frequency), dietary diversity, diet quality, school enrolment status, school completion, and cognitive, maths, and/or language skills	Not applicable
Study design	Randomized controlled trials, non-randomized controlled trials including controlled before-after studies	Non-randomized trials including controlled before-after studies that did not account for baseline differences, observational studies including cohort, case–control, and cross-sectional designs, and editorial commentaries, opinions, and review articles

### Eligibility criteria

- Types of studies: We will include randomized controlled trials (RCT), with the intervention randomized to individuals or in clusters (including classes, schools, and groups/clubs), non-RCTs including controlled before-after studies that have reported interventions to address any form of adolescent malnutrition and/or educational outcomes when compared to a control group. We will consider non-RCTs as those in which the investigator controls allocation, which is not random. Controlled before-after studies would be those including pre- and post-intervention assessment of outcomes as well as non-random group allocations that are not controlled by the researchers. Non-RCTs including controlled before-after studies will be eligible for inclusion, provided the baseline differences between study arms are accounted for in the analysis.
- Types of participants: Studies involving adolescents (boys and girls) aged 10 to 19 years.
- Study settings: Studies conducted in LMICs—as defined by the World Bank in the year 2020 [34].
- Types of interventions: Studies involving interventions for one or more of the following: nutrition education, physical education, interventions to promote healthy diets, interventions promoting physical activity, school gardens, school food and nutrition policies, school environment interventions, and water sanitation and hygiene (WASH) interventions. We will consider those interventions as multi-component, which include two or more above-mentioned components [8, 28].
- Comparison intervention: The control (comparison) in each included study can be participants who did not receive any intervention, received standard health education and/or physical education, or any other intervention in the school setting [28, 31].
- Outcome measures: The primary outcomes will be body mss index (BMI) z scores and school attendance. The secondary outcomes will include a change in anthropometry (e.g., height and weight status, BMI, height-for-age z scores, weight-for-age z scores, weight-for-height z scores, skin-fold thickness measures, stunting, underweight, wasting, body mass index, overweight, obesity, waist-to-height ratio, and central obesity), knowledge of diet and nutrition, dietary intake (i.e. amount and frequency), dietary diversity, and diet quality. The education-related secondary outcomes will include school enrolment status, school completion, and cognitive, maths, and/or language skills.
- We will include published articles and ongoing studies for which preliminary findings are available.
- We will not place any restrictions in terms of the year of publication, language, sample size of the study, or duration of the intervention provided.

### Exclusion criteria

We will not consider the following studies:

- Non-RCTs and controlled before-after studies that did not account for the baseline differences between the study arms;
- Studies without a proper control (comparison) group/arm; e.g., uncontrolled before-after studies,
- Observational studies including cohort, case–control, and cross-sectional designs,
- Editorial commentaries, opinions, and review articles,
- Clinical treatments/interventions targeted towards individuals with specific medical conditions such as programs intended for underweight, overweight, or obese adolescents,
- Studies of only school feeding and interventions providing micronutrients through fortification & targeted supplementation as these are covered by other reviews in the field, and
- Studies of educational interventions such as focusing on building relationships, classroom environment and/or management, and behavioral and learning interventions focusing *only* on educational outcomes (e.g., school or classroom participation, classroom environment, and teacher performance).

### Information sources

The following principal sources of electronic reference libraries will be searched: Medline (through PubMed), Embase, CENTRAL (through the Cochrane Library), CINAHL (Cumulative Index to Nursing and Allied Health Literature), and Google Scholar. All databases will be searched for eligible studies from the inception of each database through September 2020. Detailed examination of cross-references and bibliographies of included studies to identify additional sources of information will also be performed. This search of studies will be supplemented by reviewing organizational websites such as the World Food Programme, World Bank, Food and Agriculture Organization, United Nations Children’s Fund, and United Nations Population Fund. Colleagues who are native speakers, whenever possible will translate articles written in languages other than English. Studies that cannot be translated into English language will be excluded.

### Search strategy

Guided by the conceptual framework, a broad search strategy (e.g. type of study [randomized controlled trial OR controlled before-after studies OR quasi-experimental studies] AND intervention domain [nutrition] AND population [adolescents] AND setting [low- and middle-income countries]) will be performed in PubMed without time restrictions. We consulted with a health science librarian to develop the PubMed search strategy, which is provided in Additional File 1. The sensitivity of the search strategy will be examined by confirming that several sentinel articles are identified. The PubMed strategy will be adapted to the syntax appropriate for other databases. The following details will be documented for each search: databases searched, date of search conducted on, search strategy (i.e., subject headings and keywords used, including whether terms are exploded, truncated and how terms are combined),—filters used, number of results retrieved for each search, the total number of records, duplicates identified, and numbers pre- and post-screening. Also, all publications identified through hand searching will be noted with a source (i.e., name of journal/website, conference proceedings, etc.) and the years.

### Data Management

EndNote X9 (Clarivate Analytics, PA, USA) will be used to store the records retrieved from searches of electronic databases. The records will also be imported into Covidence (Veritas Health Innovation, Melbourne, Australia), an internet-based program that facilitates the streamlined management of the systematic review. Duplicate records will be detected and removed first by EndNote and then by Covidence.

Two reviewers will independently assess the search results based on the inclusion and exclusion criteria. First, all searched titles and abstracts will be reviewed to exclude irrelevant studies. Disagreements between the two reviewers will be resolved by discussion or by a third reviewer, if necessary.

A study flow diagram will be maintained as recommended by the PRISMA statement (Preferred Reporting Items for Systematic Reviews and Meta-Analyses) [35], with the specific reasons for excluding studies. Neither of the reviewers will be blind to journal titles or the names of the authors.

### Data extraction

Two reviewers will independently extract and enter the data of studies included in the review. A data extraction form provided as Additional File 2, will be developed and then pilot-tested on five randomly selected studies. The following information will be extracted for each selected study: title, authors (first author and corresponding author), contact information of corresponding author, journal (or source for reports), year of publication, year of intervention implementation, country and geographical setting, study design, sample size (if a cluster randomised trial, number of clusters and average cluster size), sample characteristics (e.g., age, sex, and socioeconomic status of the participants), intervention (including duration, intervention type, guiding theory/framework, intervention description, delivery mechanisms and agents, and procedures employed for selection, training and supervision of delivery agents, intervention coverage and fidelity information, and challenges and barriers encountered in intervention delivery), measure of adherence, information on control/comparison intervention, outcomes assessed, time-points, main findings with point estimates and measures of variance (standard errors, 95% CI and/or *p*-values), and reasons provided for success/failure. Multiple reports of a single study will be collated as additional results may be provided in different reports. In case of missing information or inconsistent results across reports of a single study, we will contact the corresponding author via email to obtain more accurate results or additional information. A maximum of two contact attempts will be made. If we cannot resolve the issues with the data after contacting the authors, we will analyze the available data and discuss the possible impact of the missing data.

### Risk of bias assessment

For the assessment of the risk of bias of the selected studies, we will use the Cochrane Collaboration’s revised tool for assessing the risk of bias in randomized trials [36]. Two reviewers will independently evaluate methodological quality. Any uncertainties or disagreements will be resolved by discussion or by a third reviewer, whenever needed. The tool is a domain-based evaluation, in which critical assessments will be made separately for the bias arising from the randomization process, the bias due to deviation from intended interventions, the bias due to missing outcome data, the bias in the measurement of the outcome, and selective outcome reporting. The judgment for each entry will involve assessing the risk of bias as “low risk,” as “high risk,” or as “some concerns,” with the last category indicating either lack of information or uncertainty about the potential for bias.

We will use the Risk of Bias in Non-randomized Studies of Interventions (ROBINS-I) tool [37], to assess the risk of bias for controlled before-after studies and non-randomized controlled trials. This tool considers biases from confounding, the bias in selection of participants into the study, the bias in classification of interventions, bias due to deviations from intended interventions, the bias due to missing data, the bias in the measurement of outcomes, and the bias in selection of the reported results. Each domain will be judged as “low risk of bias,” “moderate risk of bias,” “serious risk of bias,” “critical risk of bias,” or “no information.” Based on the domain-specific judgments, we will consider a non-randomized study (a) at low risk of bias if it is judged to have a low or moderate risk of bias for all domains; or (b) at high risk of bias if it is judged to have a serious or critical risk of bias in one or more domains; or (c) have some concerns if the assessment is unclear for one or more domains but low or moderate for all other domains. We will contact the corresponding authors of the reports to obtain more information, when necessary. We will summarize the results of the assessment of the risk of bias in a table, in which the judgment for each domain will be presented with a justification.

We will analyze the overall strength of the evidence for each outcome using the Grading of Recommendations Assessment, Development and Evaluation (GRADE) tool [38].

### Synthesis of evidence

A systematic synthesis of all included studies will be presented in the text as well as in a table, using the SWiM guidelines (Synthesis Without Meta-analysis) [39]. The synthesis will report on the grouping of studies, a standardized metric for each outcome, synthesis methods, criteria used to prioritize results for summary, heterogeneity in effects, the certainty of the evidence, data presentation methods, reporting of results, and limitation of synthesis. Based on our conceptual framework, the interventions will be identified by an iterative process of data collating and key findings will be broken down into specific categories, derived from the articles. The synthesis will also take into account the different comparisons included for grouping interventions and study designs. For multi-component interventions, we will analyze and summarize the effectiveness findings based on dominant intervention components. We will not conduct the meta-analyses given the considerable heterogeneity of both the interventions and outcomes.

We will follow the Preferred Reporting Items for Systematic Reviews and Meta-Analyses (PRISMA) checklist and guidelines to ensure a robust and replicable process [40]. Effect estimates for continuous outcomes will be expressed as mean differences (with 95% CI) comparing the intervention group with the control group; effect estimates for dichotomous outcomes will be expressed as risk ratios, rate ratios, hazard ratios, or odds ratios (all with 95% CI), comparing the intervention group with the control group.

### Registration and reporting

This systematic review protocol has been registered on the PROSPERO database (CRD42020211109), based on the Preferred Reporting Items for Systematic Reviews and Meta-Analyses Protocols (PRISMA) statement guidelines. In the event of protocol amendments, the date of each amendment will be accompanied by a description of each change and the rationale on PROSPERO.

This protocol is written following the Preferred Reporting Items for Systematic Review and Meta-Analysis Protocols (PRISMA-P) [40]. The PRISMA-P checklist can be obtained from Additional File 3**.** We will report this systematic review following the SWiM guideline [39] and *Cochrane Handbook for Systematic Reviews of Interventions* [41].

## Discussion

Combating malnutrition in all its forms is one of the greatest global health challenges influenced by economic and income growth, urbanization and globalization, and related shifts in the quality and quantity of human diets. In 2016, the United Nations Decade of Action on Nutrition for the period 2016 − 2025 came to life [42], calling for specific coordinated actions through cross-cutting and coherent policies, programs, and initiatives to address increasing DBM. As the global community transitions from a predominant focus on the eradication of severe and acute undernutrition within the framework of the Millennium Development Goals (MDGs) to the broader nutrition focus of the Sustainable Development Goals (SDGs), including all forms of malnutrition and noncommunicable diseases, addressing DBM offers an unexplored window of opportunity for integrated actions. The agenda for Sustainable Development 2030 has also identified a need for tracking indicators to meet targets, especially for the Global Strategy for Women’s, Children’s and Adolescents’ Health [30]. In this review, we will focus on DBM in adolescents for multiple reasons. First, adolescence is a critical period for growth and development, with higher nutritional demands placing adolescents at greater risks of malnutrition. Second, the DBM can manifest at the individual, family, and community, region, or country levels [7]. Especially, the stable rates of undernutrition in many LMICs coupled with the dramatic increases in overweight, obesity, and associated non-communicable diseases are placing heavy tolls on individuals, families, economies, and healthcare systems [43]. Third, after infancy, growth during adolescence is faster than any other period of life. Adolescents experience both growth and development in their skeletal system as well as their brain during adolescence. Therefore, the adolescence period offers a unique chance to address nutritional problems and develop healthy and long-lasting dietary and lifestyle habits [6]. Fourth, given that approximately 16 million girls between the ages of 15 and 19 years enter motherhood every year across the globe, their nutritional status is important not only for their health but also for the health of their newborn as well as the family [44]. Finally, good nutrition is one of the prerequisites for effective learning and vice versa [6, 8]. Research in the past two decades has shown that social determinants of health are powerful and interconnected, especially with nutrition, brain functions, cognitive development, and educational performance, and that disparity in any of them is exacerbating the others and accumulating over time [45].

Several evidence-informed actions exist to address adolescent nutrition including school-based nutrition interventions. These interventions can be broadly identified as 1) promoting healthy diets through education; 2) providing additional micronutrients through fortification of staple foods and targeted supplementation; 3) managing acute malnutrition; 4) promoting physical activity; and 5) providing access to a safe environment and hygiene [44, 45]. These interventions can be broadly classified into two categories: 1) discrete single-component interventions; and 2) integrated interventions. Single-component interventions include obesity prevention, anemia prevention, and micronutrient supplementation whereas the integrated interventions integrate two or more intervention components into a single intervention. The most widely recognized example of the integrated school-based health and nutrition intervention is World Health Organization’s (WHO) Health Promoting Schools (HPS) framework [46] which recognizes the link between health, nutrition, and education and encourages a whole school approach to improving health and educational outcomes. In many LMICs, school-based integrated nutrition programs, consisting of promoting healthy eating, nutrition education, and physical activity have been increasingly implemented to address the multiple forms of malnutrition among school-aged children [47].

Several systematic reviews of single-component interventions on high-income and/or LMICs suggest promising but modest evidence of an effect, with these reviews examining a range of interventions such as school feeding, nutrition education, obesity prevention, and physical activity [10–20]. For example, a systematic review of 50 school-based obesity prevention interventions found significant differences between groups on BMI and BMI *z* − score [16]. A systematic review of 18 studies on school feeding interventions found beneficial effects in terms of gained weight among the participants from lower-income countries, and improved performances in cognitive tasks [10]. However, these single risk domain interventions fail to recognize that multiple lifestyle risk behaviors in adolescents co-occur as clusters and track into adulthood [8]. It is also possible that discrete interventions that are not coordinated could be ineffective [48] as they often lack sufficient buy-in, training, and fidelity and are less likely to be sustained [49]. Given the limited availability of funding for prevention and treatment interventions for adolescents, the Lancet Commission on Adolescent Health [8] and WHO’s Global Accelerated Action for the Health of Adolescents guideline [50] highlight the necessity of synchronized prevention efforts to target multiple health risk behaviors in adolescents.

Several literature reviews and systematic reviews published in the last two decades examine the effectiveness of integrated school-based health and nutrition interventions [21–29]. A cross-national Cochrane review of 67 clustered randomized control trial (CRCT) studies on the effectiveness of HPS initiatives observed positive improvements in BMI, physical activity, and nutrition of the participants in a few studies [25]. In another systematic review of 11 multi-strategy nutrition education interventions, four studies reported significant improvements in anthropometric measures and nine studies showed significant changes in dietary intakes [28]. Recent research on interventions targeting multiple risk behaviors in school settings also suggests that by improving health outcomes, these programs can also enhance educational outcomes such as student attendance, school engagement, classroom behavior, mood, concentration, memory, standardized test scores, grade point average, grade advancement, and high school completion [51–55]. However, the impact of integrated school-based nutrition interventions on academic outcomes and cognitive development is modest [24]. Evidence on the involvement of family or community in school-based health interventions is mixed [25]. Overall, evidence suggests that comprehensive, multicomponent school-based interventions hold greater potential in promoting and supporting positive health behavior changes in the long term than single-component nutrition interventions.

Although integrated school-based nutrition interventions are promising, there are several gaps in the available evidence. First, most of the integrated school-based health and nutrition interventions focused on general health and wellbeing, healthy eating, and physical activity and lacked attention to multiple forms of malnutrition [25]. Second, these reviews have not examined which components of the package or the characteristics of the context contributed to the reported effect. Third, much of the above-mentioned literature on integrated school-based health intervention does not focus only on adolescents and includes child-adolescent binomial. This is a problem because it combines the two life stages into one and tends to neglect the unique health and development challenges of adolescents. Fourth, most of the studies included in these reviews come from high-income countries. Thus, little is known about the impacts of integrated interventions on specific nutrition and educational outcomes of adolescents in LMICs. Finally, previous reviews of integrated school-based interventions are outdated, with the most recent review published in 2015 [25], thus do not reflect all of the currently available evidence. Therefore, an updated and refined synthesis of evidence on school-based nutrition interventions that target multiple forms of malnutrition and educational outcomes of adolescents, engage students, families, and communities, and enable education, health and nutrition, and other agencies to synergistically improve nutritional status and education outcomes of all adolescents, is warranted.

We anticipate that the findings of this review will help advance the application of recommendations noted in the Lancet Commission on Adolescent Health [8], WHO’s Global Accelerated Action for Health of Adolescents [50], and Child and Adolescent Health Volume of the third edition of Disease Control Priorities [6] advocate for integrated school-based health and nutrition approaches. Results of this review may contribute to the formulation of future programs to address the immediate and growing needs of school-going children as well as approaches to link schools, families, and the wider community through such interventions. Further, these findings may also aid policymakers, researchers, practitioners, and government and non-governmental agencies in developing and implementing interventions to improve integrated health, address multiple forms of malnutrition, and educational outcomes for school-age children in LMICs.

## Supplementary Information

**Additional file 1.** COREQ checklist. Consolidated criteria for reporting qualitative studies (COREQ): 32-item checklist.

**Additional file 2.** Questionnaire. Questionnaire that was used for background characteristics family caregivers.

**Additional file 3.** Topic list for semi-structured interviews. Topic list that was used in this study.

## Data Availability

All data that will be generated and analyzed during this study will be included in the published article or its supplementary information files.

## Abbreviations

BMI: Body Mass Index
CI: Confident interval
CINAHL: Cumulative Index to Nursing and Allied Health Literature
CRCT: Clustered randomized control trial
DBM: Double burden of malnutrition
HPS: Health Promoting Schools
LMIC: Low- and middle-income country
MDGs: Millennium Development Goals
PICOS: Population, Intervention, Comparison, Outcome, Study design
PRISMA: Preferred Reporting Items for Systematic Reviews and Meta-Analyses
PRISMA-P: Preferred Reporting Items for Systematic Review and Meta-Analysis Protocols
PROSPERO: International Prospective Register of Systematic Reviews
RCT: Randomized controlled trial
ROBINS-I: Risk of Bias in Non-randomized Studies of Interventions
SDGs: Sustainable Development Goals
SWiM: Synthesis Without Meta-analysis
WHO: World Health Organization
