# Supplementary material for: School-based interventions targeting double burden of malnutrition and educational outcomes of adolescents in low- and middle-income countries: protocol for a systematic review
Source: Syst Rev. 2021 Jul 10;10:204. doi: 10.1186/s13643-021-01756-9 (PMC8272909; doi:10.1186/s13643-021-01756-9)
Supplement: Supplementary file 1 — Additional file 1. COREQ checklist. Consolidated criteria for reporting qualitative studies (COREQ): 32-item checklist. [file 13643_2021_1756_MOESM1_ESM.docx]

**Additional File 1:** PubMed Search Strategy

| **No.** | **Concept** | **PubMed search terms** |
| --- | --- | --- |
| #1 | **Randomized controlled trial**  **Controlled before-after studies**  **Quasi experimental studies** | ("randomized controlled trial"[pt] OR "controlled clinical trial"[pt] OR "clinical trials as topic"[mesh] OR "random allocation"[mesh] OR "double-blind method"[mesh] OR "single-blind method"[mesh] OR "clinical trial"[pt] OR "research design"[mesh:noexp] OR "comparative study"[pt] OR "evaluation studies"[pt] OR "follow-up studies"[mesh] OR "prospective studies"[mesh] OR "cross-over studies"[mesh] OR "Controlled Before-After Studies"[Mesh] OR "clinical trial"[tw] OR ((singl*[tw] OR doubl*[tw] OR trebl*[tw]) AND (mask*[tw] OR blind*[tw])) OR placebo*[tw] OR quasi experiment*[tiab] OR quasiexperiment*[tiab] OR random*[tw] OR "control"[tw] OR "controls"[tw] OR prospectiv*[tw] OR volunteer*[tw]) |
| #2 | **Nutrition and health interventions**  (Dietary supplements, healthy diet, healthy eating, healthy nutrition, overweight, anaemia, obesity, weight control, weight management, micronutrient supplementation diet/nutrition education, school meal, physical activity, school garden, and WASH, and nutrition policy) | **("Health Education"[Mesh:NoExp] OR "Health Promotion"[Mesh] OR "Adolescent Health Services"[Mesh] OR "Preventive Health Services"[Mesh:NoExp] OR preventive health[tiab] OR ("Dietary Supplements"[Mesh] OR "Diet, Healthy"[Mesh] OR "Fruit"[Mesh:NoExp] OR "Vegetables"[Mesh] OR "Meals"[Mesh] OR "Anemia"[Mesh] OR "Overweight"[Mesh] OR anemia[tiab] OR anaemia[tiab] OR healthy diet*[tiab] OR healthy eating[tiab] OR healthy food*[tiab] OR healthy nutrition*[tiab] OR nutrition counsel*[tiab] OR nutritional counsel*[tiab] OR obesity[tiab] OR over weight[tiab] OR overweight[tiab] OR weight control[tiab] OR weight management[tiab] OR "Malnutrition"[Mesh] OR "malnutrition"[tiab] OR "undernutrition"[tiab] OR "undernutritional"[tiab] OR "undernourished"[tiab] OR "undernourishment"[tiab] OR "wasting"[tiab] OR "wasted"[tiab] OR "stunting"[tiab] OR "stunted"[tiab] OR "thinness"[MeSH] OR "thinness"[tiab] OR "underweight"[tiab] OR "underweights"[tiab] OR breakfast*[tiab] OR contraception education*[tiab] OR contraceptive education*[tiab] OR depressive[tiab] OR diet education[tiab] OR dietary education[tiab] OR dietary intervention[tiab] OR dietary supplement*[tiab] OR folic acid supplement*[tiab] OR fruit[tiab] OR fruits[tiab] OR health education[tiab] OR health intervention[tiab] OR health promotion[tiab] OR healthy eating[tiab] OR healthy food*[tiab] OR healthy diet*[tiab] OR lunch*[tiab] OR meals[tiab] OR micronutrient supplement*[tiab] OR nutrient supplement*[tiab] OR nutrition education[tiab] OR nutrition intervention[tiab] OR nutritional education[tiab] OR nutritional intervention[tiab] OR nutritional supplement*[tiab] OR school meal*[tiab] OR snack*[tiab] OR vegetable*[tiab] OR vitamin supplement*[tiab] OR "exercise"[MeSH] OR "exercise" [tiab] OR ("physical"[tiab] AND "activity"[tiab]) OR "physical activity"[tiab] OR garden*[tiab] OR "WASH" [tiab]) OR "hygiene"[MeSH] OR "hygiene"[tiab] OR "sanitation"[MeSH] OR "sanitation"[tiab] OR "nutrition policy"[MeSH] OR ("nutrition"[tiab] AND "policy"[tiab]) OR "nutrition policy"[tiab]) AND ("School Health Services"[Mesh] OR "Schools"[Mesh:NoExp] OR government school*[tiab] OR high school*[tiab] OR highschool*[tiab] OR junior high[tiab] OR middle school*[tiab] OR private school*[tiab] OR public school*[tiab] OR school based[tiab] OR school breakfast*[tiab] OR school day[tiab] OR school health[tiab] OR school lunch*[tiab] OR school meal*[tiab] OR school nutrition*[tiab] OR school setting[tiab] OR schoolday[tiab] OR secondary school*[tiab])** |
| #3 | **Adolescents** | ("Adolescent"[Mesh] OR adolescent[tiab] OR adolescents[tiab] OR adolescence[tiab] OR teen[tiab] OR teens[tiab] OR teenage*[tiab] OR high school*[tiab] OR highschool*[tiab] OR middle school*[tiab] OR junior high*[tiab] OR preadolescen*[tiab] OR prepupert*[tiab] OR pubert*[tiab] OR secondary school*[tiab] OR pubescen*[tiab] OR youth[tiab] OR youths[tiab] OR young people[tiab] OR 10 years old[tiab] OR 11 years old[tiab] OR 12 years old[tiab] OR 13 years old[tiab] OR 10 years of age[tiab] OR 11 years of age[tiab] OR 12 years of age[tiab] OR 13 years of age[tiab] OR 3rd grade*[tiab] OR 4th grade*[tiab] OR 5th grade*[tiab] OR 6th grade*[tiab] OR age 10[tiab] OR age 11[tiab] OR age 12[tiab] OR age 13[tiab] OR aged 10[tiab] OR aged 11[tiab] OR aged 12[tiab] OR aged 13[tiab] OR age ten[tiab] OR age eleven[tiab] OR age twelve[tiab] OR age thirteen[tiab] OR grade 3[tiab] OR grade 4[tiab] OR grade 5[tiab] OR grade 6[tiab] OR grades 3[tiab] OR grades 4[tiab] OR grades 5[tiab] OR grades 6[tiab] OR third grade*[tiab] OR fourth grade*[tiab] OR fifth grade*[tiab] OR sixth grade*[tiab]) |
| #4 | **Low- and middle-income countries** | ("Developing Countries"[Mesh] OR developing countr*[tiab] OR under developed countr*[tiab] OR lmic*[tiab] OR ((less developed[tiab] OR low income[tiab] OR lower income[tiab] OR low and middle income[tiab] OR low middle income[tiab] OR resource poor[tiab] OR resource constrained[tiab] OR low resource[tiab] OR limited resource*[tiab] OR resource limited[tiab]) AND (country[tiab] OR countries[tiab] OR region[tiab] OR regions[tiab] OR setting*[tiab] OR area[tiab] OR areas[tiab])) OR "Africa South of the Sahara"[Mesh] OR "Central America"[Mesh] OR "South America"[Mesh] OR "Latin America"[Mesh] OR "Caribbean Region"[Mesh] OR "Mexico"[Mesh] OR "Asia"[Mesh:NoExp] OR "Asia, Central"[Mesh] OR "Asia, Northern"[Mesh] OR "Asia, Southeastern"[Mesh] OR "Asia, Western"[Mesh] OR "China"[Mesh] OR "Korea"[Mesh] OR "Mongolia"[Mesh] OR Afghan*[tiab] OR Africa[tiab] African[tiab] OR Algeria*[tiab] OR American Samoa*[tiab] OR Angola*[tiab] OR Argentin*[tiab] OR Bangladesh*[tiab] OR Barbad*[tiab] OR Belorussian[tiab] OR Beliz*[tiab] OR Benin*[tiab] OR Bhutan*[tiab] OR Bolivia*[tiab] OR Botswan*[tiab] OR Brazil*[tiab] OR "Burkina Faso"[tiab] OR Burkinabe[tiab] OR Burund*[tiab] OR Cambodia*[tiab] OR Cameroon*[tiab] OR "Cape Verde"[tiab] OR "Cape Verdean"[tiab] OR "Central African Republic"[tiab] OR Chad*[tiab] OR Chile*[tiab] OR China[tiab] OR Chinese[tiab] OR Colombia*[tiab] OR Comoros[tiab] OR Comorian[tiab] OR Congo[tiab] OR Congolese[tiab] OR Costa Rica*[tiab] OR "Côte d’Ivoire"[tiab] OR “Ivory Coast”[tiab] OR Ivorian[tiab] OR Croatia*[tiab] OR Croat[tiab] OR Cuba*[tiab] OR Djibouti*[tiab] OR Dominica*[tiab] OR "Dominican Republic"[tiab] OR Ecuador*[tiab] OR Egypt*[tiab] OR "El Salvador"[tiab] OR Salvadorian[tiab] OR "Equatorial Guinea"[tiab] OR Guinean[tiab] OR Eritrea*[tiab] OR Ethiopia*[tiab] OR Fiji*[tiab] OR Gabon*[tiab] OR Gambia*[tiab] OR Gaza[tiab] OR Gazan[tiab] OR Ghana[tiab] OR Ghanaian[tiab] OR Grenad*[tiab] OR Guatemala*[tiab] OR Guinea[tiab] OR Guyan*[tiab] OR Haiti*[tiab] OR Hondura*[tiab] OR Hungar*[tiab] OR India[tiab] OR Indian[tiab] OR Indonesia*[tiab] OR Iran*[tiab] OR Iraq*[tiab] OR Jamaica*[tiab] OR Jordan*[tiab] OR Kenya[tiab] OR Kenyan[tiab] OR Kiribati[tiab] OR Korea*[tiab] OR Kyrgy*[tiab] OR Laos[tiab] OR Laotian*[tiab] OR Lebanon[tiab] OR Lebanese[tiab] OR Lesotho[tiab] OR Liberia*[tiab] OR Libya*[tiab] OR Macedonia*[tiab] OR Madagasca*[tiab] OR Malawi*[tiab] OR Malaysia*[tiab] OR Maldives[tiab] OR Maldivian[tiab] OR Mali[tiab] OR Malian*[tiab] OR "Marshall Islands"[tiab] OR Mauritania*[tiab] OR Mauritius[tiab] OR Mauritian[tiab] OR Mayotte[tiab] OR Mexic*[tiab] OR Micronesia*[tiab] OR Moldov*[tiab] OR Mongolia*[tiab] OR Morocc*[tiab] OR Mozambique[tiab] OR Mozambican[tiab] OR Myanmar[tiab] OR Namibia*[tiab] OR Nepal*[tiab] OR Nevis[tiab] OR Nicaragua*[tiab] OR Niger*[tiab] OR "Northern Mariana Islands"[tiab] OR Oman*[tiab] OR Pakistan*[tiab] OR Palau*[tiab] OR Panama*[tiab] OR "Papua New Guinea"[tiab] OR Paraguay*[tiab] OR Peru*[tiab] OR Philippine*[tiab] OR Filipino*[tiab] OR Poland[tiab] OR Polish[tiab] OR Rwanda*[tiab] OR Samoa*[tiab] OR Sao Tome*[tiab] OR Principe[tiab] OR Senegal*[tiab] OR Seychell*[tiab] OR Sierra Leon*[tiab] OR Solomon Island*[tiab] OR Somali*[tiab] OR South Africa*[tiab] OR Sri Lanka*[tiab] OR "Saint Kitts"[tiab] OR "St Kitts"[tiab] OR "Saint Lucia"[tiab] OR "St Lucia"[tiab] OR "Saint Vincent" [tiab] OR "St Vincent"[tiab] OR Sudan*[tiab] OR Suriname*[tiab] OR Swaziland Or Swazi[tiab] OR Syria*[tiab] OR Tajik*[tiab] OR Tanzania*[tiab] OR Thailand[tiab] OR Thai[tiab] OR "Timor Leste"[tiab] OR Togo*[tiab] OR Tonga*[tiab] OR Trinidad[tiab] OR Trinidadian[tiab] OR Tobago[tiab] OR Tobagonian[tiab] OR Tunisia*[tiab] OR Turky[tiab] OR Turkish[tiab] OR Uganda*[tiab] OR Uruguay*[tiab] OR Vanuat*[tiab] OR Venezuela*[tiab] OR Vietnam*[tiab] OR "West Bank"[tiab] OR Yemen*[tiab] OR Zambia*[tiab] OR Zimbabwe*) |
