# Supplementary material for: School-based interventions targeting double burden of malnutrition and educational outcomes of adolescents in low- and middle-income countries: protocol for a systematic review
Source: Syst Rev. 2021 Jul 10;10:204. doi: 10.1186/s13643-021-01756-9 (PMC8272909; doi:10.1186/s13643-021-01756-9)
Supplement: Supplementary file 2 — Additional file 2. Questionnaire. Questionnaire that was used for background characteristics family caregivers. [file 13643_2021_1756_MOESM2_ESM.docx]

**Additional File 2 Data extraction form**

| **Title & author** | **Journal /source** | **Calendar year of publication** | **Calendar year of study** | **Country** | **Study design** | **Sample size** | **Sample characteristics (age, sex, socioeconomic status, etc.)** | **Timing of intervention** | **Duration of intervention** | **Guiding theory/ framework** | **Content & components of the intervention** | **Intervention delivery mechanism and agents** | **Selection, training, and supervision of delivery agents** | **Intervention coverage** | **Measure of adherence** | **Control group** | **Outcomes and time points** | **Main findings** | **Theory to explain the success** | **Theory to explain the failure** |
| --- | --- | --- | --- | --- | --- | --- | --- | --- | --- | --- | --- | --- | --- | --- | --- | --- | --- | --- | --- | --- |
|  |  |  |  |  |  |  |  |  |  |  |  |  |  |  |  |  |  |  |  |  |
|  |  |  |  |  |  |  |  |  |  |  |  |  |  |  |  |  |  |  |  |  |
|  |  |  |  |  |  |  |  |  |  |  |  |  |  |  |  |  |  |  |  |  |
|  |  |  |  |  |  |  |  |  |  |  |  |  |  |  |  |  |  |  |  |  |
|  |  |  |  |  |  |  |  |  |  |  |  |  |  |  |  |  |  |  |  |  |
|  |  |  |  |  |  |  |  |  |  |  |  |  |  |  |  |  |  |  |  |  |
|  |  |  |  |  |  |  |  |  |  |  |  |  |  |  |  |  |  |  |  |  |
